# Lectures on the Malarial Fevers

**Published:** 1898-07

**Authors:** 


Lectures on the Malarial Fevers. By William Sydney Thayer, M. D., Associate Professor of Medicine in Johns-Hopkins University. D. Appleton & Co., New York. 1897. Pages 319.

This work appears to be a reproduction of the ordinary course of lectures delivered on this subject, to the students at Johns-Hopkins. It constitutes a very thorough exposition of the present knowledge of malaria, especially its usual forms. A complete exposition of the methods and results of microscopical examinations of the blood is a feature, which adds much to the value of the work. Also a critical study of the discovery of the plasmodium malariæ by Laveran, and the developments along this line, up to the present time, is another very valuable feature. It contains the best article on malarial hæmoglobinuria that we have read, though, we are bound to disagree with the author in some respects. As usual with those who derive their knowledge exclusively from laboratory and hospital experience, the author knows nothing but quinine in the treatment, barely mentioning arsenic, and only referring to purgatives to disapprove of them. The work is altogether the best treatise on malaria that we have read recently. J.

